# Cross-validation of the high-capacity tensiometer and thermocouple psychrometer for continuous monitoring of xylem water potential in saplings

**DOI:** 10.1093/jxb/erab412

**Published:** 2021-09-10

**Authors:** Roberta Dainese, Bruna de CFL Lopes, Giuseppe Tedeschi, Laurent J Lamarque, Sylvain Delzon, Thierry Fourcaud, Alessandro Tarantino

**Affiliations:** 1 Department of Civil and Environmental Engineering, University of Strathclyde, Glasgow, UK; 2 CIRAD, UMR AMAP, Montpellier, France; 3 AMAP, Univ. Montpellier, CIRAD, CNRS, INRAE, IRD, Montpellier, France; 4 Politecnico di Bari, Bari, Italy; 5 Univ. Bordeaux, INRAE, UMR BIOGECO, Pessac, France; 6 Département des Sciences de l’Environnement, UQTR, Trois-Rivières, Québec, Canada; 7 CNRS Aix-Marseille University, France

**Keywords:** High-capacity tensiometer, pressure chamber, thermocouple psychrometer, water status monitoring, water tension, xylem water potential

## Abstract

The pressure chamber, the most popular method used to measure xylem water potential, is a discontinuous and destructive technique and is therefore not suitable for automated monitoring. Continuous non-destructive monitoring could until very recently be achieved only by use of the thermocouple psychrometer (TP). Here we present the high-capacity tensiometer (HCT) as an alternative method for continuous non-destructive monitoring. This provided us with a unique chance to cross-validate the two instruments by installing them simultaneously on the same sapling stem. The HCT and the TP showed excellent agreement for xylem water potential less than –0.5 MPa. Response to day/night cycles and watering was remarkably in phase, indicating excellent response time of both instruments despite substantially different working principles. For xylem water potential greater than –0.5 MPa, the discrepancies sometimes observed between the HCT and TP were mainly attributed to the kaolin paste used to establish contact between the xylem and the HCT, which becomes hydraulically poorly conductive in this range of water potential once dried beyond its air-entry value and subsequently re-wetted. Notwithstanding this limitation, which can be overcome by selecting a clay paste with higher air-entry value, the HCT has been shown to represent a valid alternative to the TP.

## Teaser

The high-capacity tensiometer and thermocouple psychrometer measure xylem water potential with high accuracy and adequate response time, as demonstrated by simultaneous measurements on sapling stems.

## Introduction

The thermocouple psychrometer (TP) and the pressure chamber are the instruments most commonly used to measure xylem water potential. The pressure chamber is an established technique and is considered the reference for the measurement of xylem water potential. However, this technique is destructive and is therefore not suitable for continuous monitoring and/or for monitoring when a relatively small number of leaves is available, which is generally the case in laboratory experiments.

The TP developed by Dixon and Tyree (1984) has been so far the only technique available for continuous and non-destructive monitoring of xylem water potential (Martinez *et al.* 2011; Yang *et al.*, 2013; Patankar *et al.* 2013; Wang *et al.*, 2014). The TP measures xylem water potential through equilibrium via vapour phase, that is, it measures the relative humidity of the air in equilibrium with the xylem water, which is then converted to xylem water potential via the psychrometric law. Since the air acts as a semi-permeable barrier, the presence of solutes in the xylem water affects the relative humidity of the air surrounding the xylem water and, hence, the measurement by the TP (Marinho *et al.*, 2008). As a result, the TP does not allow discrimination between the osmotic and the matric components of the potential of the apoplast solution present in the xylem (Boyer, 1995). The common assumption that the osmotic component of xylem water potential is negligible (Jones, 2006) does not always hold (Campbell and Gardner, 1971; Goode and Higgs, 1973).

Like any instrument based on vapour equilibrium, the TP is sensitive to temperature fluctuations and may lose accuracy for air relative humidity close to saturation, that is, at water potential values close to zero (Bulut and Leong, 2008). In addition, the TP does not allow the measurement of water potential for the case where the xylem water pressure becomes positive, which can occur under particular conditions, for example, water-saturated soil combined with very low transpiration (Charrier *et al.*, 2017). An alternative approach to the TP consists of measuring the matric component of the xylem water potential through equilibrium via the liquid phase. Balling and Zimmermann (1990) have attempted to directly measure xylem water tension using a probe made of a capillary tube filled with water and silicone oil and inserted into a xylem vessel. The tension of the xylem water was transmitted through the liquid and measured by a pressure transducer. However, this apparatus could not record xylem water potential below –0.65 MPa (Wei *et al.*, 2001) and it was not possible to prolong measurement for more than a few hours due to cavitation occurring in the instrument.

A probe somewhat similar to the one described by Balling and Zimmermann (1990) has been developed in the field of geomechanics to measure soil water tension in the range 0–2 MPa. This probe is referred to as the high-capacity tensiometer (HCT) and it has been used extensively for almost 30 years in laboratory and field testing of unsaturated soils (Ridley and Burland, 1993; Tarantino, 2004; Marinho *et al.*, 2008). The HCT has recently been proven to be capable of measuring successfully xylem water tension by Dainese and Tarantino (2020). They tested the HCT on a chestnut tree (in the field) and pear and willow saplings (in the laboratory) and validated the HCT measurements against pressure chamber measurements.

The accuracy of the diaphragm-based pressure sensor incorporated into the HCT is typically 1–2 kPa over the entire measurement range (as inferred from the calibration in the positive range) and the effect of ambient temperature fluctuations is negligible. In addition, because the sensing diaphragm behaves symmetrically, the HCT can also measure positive xylem water pressures.

The HCT is a tool that allows the continuous and non-destructive measurement of xylem water potential. It represents an alternative to the TP and offers a unique chance to cross-validate these two instruments in terms of accuracy and response time. This paper compares the measurements made by the HCT and the TP installed simultaneously on the stem of four different saplings in the laboratory. The saplings were subjected to day/night light cycles and were tested under well-irrigated and drought conditions. The measurements made by the HCT and the TP were compared with discontinuous measurements made with the pressure chamber, used here as a reference.

### Water under tension (absolute negative pressure)

**Fig. 1.**
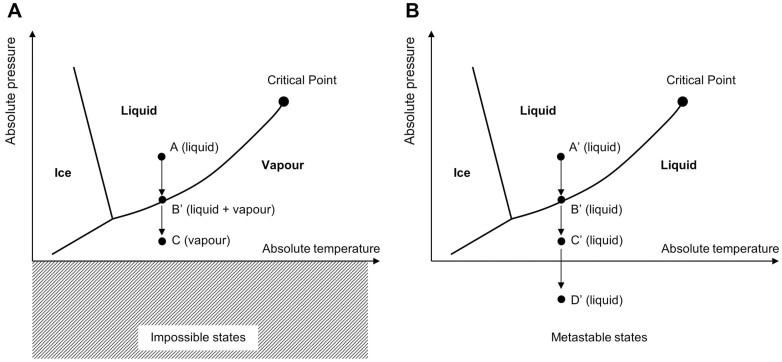
Water phase diagram. (A) Stable states. (B) Metastable states.

The traditional phase diagram of water (Fig. 1A) shows the conditions of temperature and absolute pressure characterizing the solid (ice), liquid, and vapour phases of water. Since vapour pressure cannot be negative, this diagram seems to suggest that water cannot exist in liquid phase under tension (negative absolute water pressure).

However, the phase diagram represents only the stable states of water, while other metastable states are possible without violating the principles of classic thermodynamics (Fig. 1B). The existence of a status of liquid water under tension may be considered through the van der Waals’ equation of state of fluids (De Benedetti, 1996). This equation can be used to calculate the theoretical maximum tension that can be sustained by liquid water. For example, the maximum sustainable water tension at 20 °C derived from the van der Waals’ equation of state is in the order of 100 MPa (Marinho *et al.*, 2008). However, the values of water tension measured experimentally are usually two orders of magnitude smaller than the theoretical value. The difficulty for water to reach the theoretical value is related to the presence of imperfections that lead to heterogeneous nucleation (Marinho *et al.*, 2008). Cavitation nuclei originate from air pockets that are ‘invisible to the naked eye’ that remain entrapped at the boundary between the liquid and the surface of the water container or impurities dispersed in the water.

The challenge of direct measurement of water tension is associated with its metastable state. Water under tension is subject to cavitation; that is, water tends to move from metastable states where the liquid is under tension (point D′ in Fig. 1B) to stable states where liquid and vapour phases coexist under positive absolute pressure (point B in Fig. 1A).

### Direct measurement of water tension

**Fig. 2.**
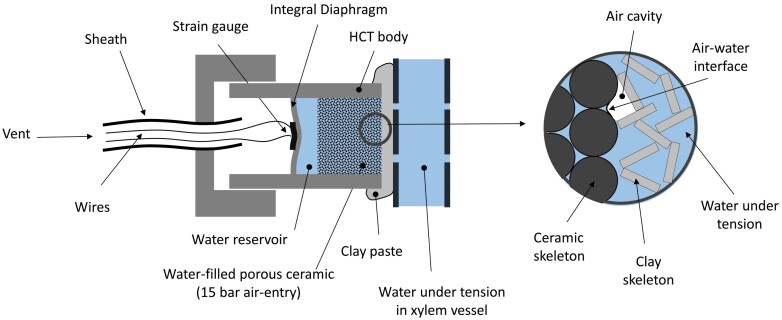
High-capacity tensiometer (after Tarantino and Mongiovì, 2002).

The transition from metastable to stable states cannot be prevented but only delayed long enough to allow the long-term measurement of water tension. This is achieved by pre-pressurizing water in the measuring instrument to dissolve the majority of cavitation nuclei (Marinho *et al.*, 2008). This is the working principle behind the HCT measurement as first developed by Ridley and Burland (1993). The typical design of the HCT includes a high air-entry porous ceramic filter, a water reservoir, and a strain-gauged diaphragm to convert water tension into an electrical signal (Fig. 2). When the instrument is applied to a sample with water under tension (negative water pressure), the water is drawn out of the water reservoir and the diaphragm bends, changing the electrical resistance of the strain gauge. The water in the reservoir and in the porous ceramic filter can sustain the water tension even if air cavities are present at the ceramic interface, which may form in the clay paste used to ensure hydraulic connection between the porous ceramic filter and the xylem vessel (Fig. 2). These menisci sustain the imbalance between water under tension in the ceramic filter and the atmospheric air pressure in these cavities. The maximum pressure imbalance, referred to as the ceramic air-entry value, is inversely proportional to the size of the largest ceramic pores and limits the maximum water tension sustainable by the HCT.

The volume of the water reservoir is generally kept very small (~4 mm^3^), as Ridley and Burland (1993) assumed that its small size was key to enable the sustained measurement of water tension. However, Mendes *et al.* (2020) have demonstrated that the volume of the reservoir does not play a critical role as generally assumed in the literature. The maximum sustainable duration of the measurement can be augmented by imposing cycles of cavitation and resaturation at high water pressure to ‘extract’ cavitation nuclei that remain undissolved upon simple pressurization (Tarantino and Mongioví, 2001). Since the porous ceramic filter does not prevent the diffusion of ions into the water reservoir (Tarantino, 2004), the measurement by the HCT is not affected by differences in concentration between the water in the instrument and the water at the measuring site, that is, the HCT measures only the matric component of xylem water potential (similar to the pressure chamber).

## Materials and methods

### Equipment

#### High-capacity tensiometer

The HCTs used in this study were manufactured according to the design developed at the University of Trento by Tarantino and Mongioví (2002). The HCT mounts a ceramic filter with a nominal air-entry value of 1.5 MPa and includes an integral strain gauge diaphragm of 0.4 mm thickness. The HCTs used in this study were calibrated in the positive range (0–1.5 MPa) at 20 °C using a dead-weight calibration device and performing a full loading–unloading cycle (0.2, 0.4, 0.8, 1.2, 2.2 MPa and reversal). A linear calibration curve was derived by best fitting the calibration data:


$$\begin{array}{l} \Psi =a_{20}+b_{20}\cdot \;mV\; \end{array}$$ (1)

where Ψ is water potential, *mV* is the electrical signal in millivolts, and *a*_20_ and *b*_20_ are the intercept and slope, respectively, of the calibration curve at 20 °C. The diaphragm-based pressure sensors showed an accuracy better than 0.003 MPa (standard deviation of the error). The calibration curve was then extrapolated to the negative range according to Tarantino and Mongioví (2003). Saturation of the ceramic filter was achieved by pre-pressurization at 4 MPa.

**Fig. 3.**
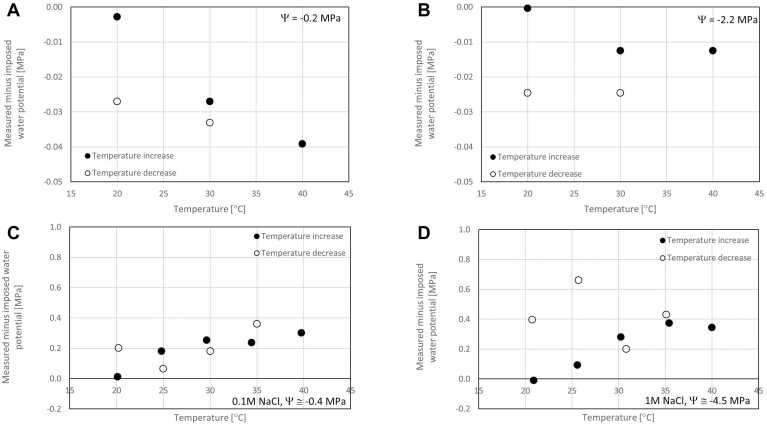
(A, B) Effect of temperature on HCT measurement: (A) imposed water potential Ψ=0.2 MPa; (B) imposed water potential Ψ=2.2 MPa. (C, D) Effect of temperature on TP measurement: (C) imposed water potential Ψ≅–0.4 MPa (0.1 M NaCl solution); (D) imposed water potential Ψ≅–4.5 MPa (1 M NaCl solution).

To investigate the effect of temperature on HCT response, calibration was repeated at 30 °C, 40 °C, back to 30 °C, and 20 °C. The error was quantified by comparing the imposed water pressure with the water pressure that would have been estimated using the calibration curve initially derived at 20 °C (see Eq. 1). This error is shown in Fig. 3A, B for two imposed water potentials, 0.2 MPa and 2.2 MPa. It can be observed that the error is significant in the sense that it is higher than the standard deviation of the error associated with the calibration at 20 °C. However, the error is relatively small (<0.04 MPa) and acceptable in most practical applications.

#### Thermocouple psychrometer

The TP used for this study is manufactured by ICT International (Armidale, NSW, Australia). To measure the relative humidity of the air in equilibrium with the xylem water, a thermocouple is cooled until the temperature drops below the dew point and a drop condenses on the thermocouple junction. Cooling is then stopped, and the drop starts evaporating into the air confined between the xylem and the instrument. The rate of evaporation is related to the relative humidity in the chamber: the higher the relative humidity, the longer it will take for the drop to evaporate (Boyer, 1995).

The response of the TP was calibrated against solutions of known relative humidity derived as shown in Supplementary Appendix S1 according to Romero (1999) (Supplementary Table S1). The thermocouple signal depends on two parameters, the cooling time and the wait time (the time lag between the start of drop evaporation and the recording of the signal).

The instrument was calibrated at 20 °C according to the procedure suggested by the manufacturer (ICT International, 2017) after setting the wait time to 6 s and the cooling time to 8 s (the same setting was maintained for the measurements). The TP was initially kept in a desiccator overnight to start from a condition of 0% relative humidity. Solutions of NaCl at different concentrations in the range of 0.1–1 mol/kg of solvent were prepared to impose known values of relative humidity. These concentrations were selected in order to cover an adequate range of xylem water potential (from –0.45 to –4.55 MPa at 20 °C).

A filter paper disk was soaked with the first solution (1 mol/kg of solvent) and placed in the disk holder provided by the manufacturer. The disk holder containing the filter paper disk was fitted to the TP. Once the reading was recorded, the disk holder was removed, a new filter paper was soaked with the second solution, placed in the disk holder, and again fitted to the TP. This procedure was repeated for the remaining four NaCl solutions up to the solution with 0.1 mol/kg of solvent. The procedure was then reversed, that is, filter paper disks with increasing molality were allowed to equilibrate with the TP.

The following equation was considered for the calibration curve, as suggested by the manufacturer:


$$\begin{array}{l} \Psi =\frac{\left(\frac{WBD}{C_{1}\cdot T_{c}+C_{2}}-CI\right)}{CS}+\frac{\Delta T}{k}\;CF_{\Delta T} \end{array}$$ (2)

where Ψ is the measured water potential, *C*_1_ and *C*_2_ are empirically derived temperature correction coefficients provided by the manufacturer, and CS and CI are the calibration slope and intercept, respectively, to be determined via calibration as instrument-specific parameters. The variables measured directly by the instrument are the psychrometric wet bulb depression, WBD (μV), the temperature of the chamber, *T*_c_ (°C), and the temperature differential between the two thermocouples of the psychrometer, Δ*T* (μV). The chromel-constantan thermocouple output (61 μV/°C) and the Δ*T* correction for *CF*_*ΔT*_ (MPa/°C) are also provided by the manufacturer.

The parameters CS and CI were determined by performing the calibration at 20 °C (in the range –0.45 to –4.55 MPa, according to Supplementary Table S1). The TP showed an accuracy lower than 0.1 MPa at 20 °C (standard deviation of the error).

To investigate the effect of temperature, a filter paper was soaked with NaCl solution at either 0.1 M or 1 M. The filter paper was placed in the disk holder and then fitted to the TP. The TP with the disk holder was placed in a climatic chamber and the temperature was increased in steps of 5 °C from 20 °C to 40 °C and then reversed. A period of 20 min was sufficient for the signal to stabilize after each temperature change.

The signal was recorded after each temperature increment or decrement and converted into water potential using Eq. (2) with the parameters CI and CS calibrated at 20 °C. The measured value was compared with the theoretical value of water potential imposed by the solution of given molality (see Supplementary Appendix S1). The error is shown in Fig. 3C, D for two imposed water pressures, –0.4 MPa and –4.5 MPa. It can be observed that the error is significant in the sense that it is higher than the standard deviation of the error associated with the calibration at 20 °C. In addition, the error is relatively large (up to 0.66 MPa) and one order of magnitude greater than the HCT.

#### Pressure chamber

The pressure chamber used in this experimental programme is manufactured by PMS Instrument Company, Albany, OR, USA (Model 1515D). It can be used to measure water tension in the range 0–10 MPa by placing a leaf inside the sealed chamber with the cut end of the petiole protruding through the seal.

### Plant material

Four broad-leaved young saplings were selected for the experiments: a cherry tree (*Prunus avium* ‘Bigarreau burlat’), an oak tree (*Quercus rubra*), a pear tree (*Pyrus communis*, Supplementary Appendix S2), and a lemon tree (*Citrus limon*). Gymnosperms were avoided because of possible clogging of the HCT porous ceramic filter by the presence of resin. The saplings (Supplementary Table S2, Supplementary Fig. S1), which were provided by an external nursery, were approximately 3–4 years old and came in pots of loose highly organic soil. Before the experiment, the saplings were kept in the laboratory under controlled temperature (20 °C) and relative humidity (50%). They were irrigated regularly and kept under a growth lamp (Sylvania Gro-Lux T8, 36 W, 3250 lumens).

### Use of the high-capacity tensiometer

#### Conditioning

To achieve adequate saturation, the porous ceramic filter of the HCT was briefly exposed to air to generate high water tension and induce cavitation in the filter. The HCT was then saturated at 4 MPa pressure for at least 48 h in a saturation chamber (Tarantino, 2004). The HCT was then removed from the saturation chamber and placed in water at atmospheric pressure. The porous ceramic filter was again exposed to air and the water tension was allowed to increase to ~1 MPa before placing the filter back into free water to release the water tension generated. This procedure was repeated twice to relieve any residual stresses in the sensing diaphragm caused by the high positive pressure applied during saturation (Tarantino and Mongioví, 2003). The HCT in free water was set to zero.

#### Application to the stem

The current version of the HCT has a diameter of 12 mm and can be installed on stems or branches with a diameter of at least 15 mm. The goal of the installation is to make the water in the xylem accessible to the instrument and avoid any localized evaporation from the contact area. The bark was removed to expose an area of xylem of approximately the same dimensions as the HCT (Supplementary Fig. S2A). The surface was then cleaned with a few drops of distilled water to remove any remaining living cells. The scratching procedure was the same as that used for the TP installation (ICT International, 2021). However, the exposed xylem surface was kept wet before the installation to avoid desiccation of the xylem tissues. The HCT was installed on the stem using a saturated paste of kaolin to ensure hydraulic contact between the xylem and the porous ceramic filter (Supplementary Fig. S2B). A latex membrane was then used to tightly wrap the area to avoid any evaporation from the paste (Supplementary Fig. S2C). The paste was prepared at approximately its liquid limit and was a compromise between two conflicting requirements. The water content of the paste should be sufficiently high to give enough plasticity and ensure good adherence between the HCT and the xylem. However, excessive water content would considerably increase the equilibration time, due to the amount of water that would need to be sucked out of the paste by the xylem to reach equilibrium.

#### Measurement data quality check

Following the installation of the HCT, the water tension changes very rapidly due to hydraulic equilibration between the instrument and the xylem. The saturated paste needs to lose water to the xylem until equilibrium is achieved. The HCT readings during equilibration are not representative of the water status of the plant and are discarded.

The presence of ‘stable’ air cavities in the porous ceramic filter may affect the measurement of the HCT, generating a differential between the tension in the xylem and the tension in the water reservoir of the instrument (Tarantino, 2004). For this reason, the HCT measurement is crossed-checked by installing two HCTs simultaneously on the same stem. The HCT measurement is considered to be valid if the readings of the two HCTs overlap. If the two measurements diverge, which is likely due to the ongoing expansion of air cavities in one of the two HCTs, the measurement of both HCTs is discarded (because it is generally not possible to recognize which HCT generated the faulty measurement). The need to use HCTs in pairs is consistent with the suggestion by Tarantino and Mongioví (2001) when discussing measurement in soil.

If cavitation occurs in the HCT, the measurements following cavitation are discarded because water tension is no longer transmitted to the pressure-sensing diaphragm. Cavitation is easily detected by the abrupt rise of the measured water potential to –0.1 MPa.

#### Post-measurement data quality checks

The presence of ‘stable’ air cavities in the porous ceramic filter may affect the measurement of the HCT, generating a differential between the tension in the xylem and the tension in the water reservoir of the instrument (Tarantino, 2004). The presence of spurious air cavities is checked at the end of each measurement. If the HCT does not cavitate during the measurement, it is placed in pure water and it is checked that the initial zero water potential is recovered (a residual water potential in the range from 0 to –0.02 MPa is considered acceptable according to Tarantino and Mongioví (2001). If the HCT cavitates during the measurement, it is checked that the water potential rises to –0.1 MPa upon cavitation.

### Use of the thermocouple psychrometer

The integrity of the thermocouple was assessed under a stereo microscope (Model MS100, Teslong, Irvine, CA, USA) before each installation. The installation site on the stem was prepared by removing the cork and the living tissues underneath (cambium). The exposed xylem was cleaned with few drops of distilled water and wiped dry. The TP was then installed on the stem by ensuring that one junction of the thermocouple was in contact with the xylem. The gap between the xylem and the TP was insulated with Parafilm® and silicon grease to allow the water vapour surrounding the thermocouple to achieve equilibrium with the xylem water. The cooling time was set to 8 s and the wait time to 6 s, consistent with the settings used for calibration.

### Use of the pressure chamber

Three samples of non-transpiring leaves were taken for each measurement. Each leaf was wrapped in aluminium foil and placed in a plastic bag at least 2 h before the leaf was excised and the measurement taken. When the leaf stops transpiring, the water in the leaf equilibrates with the water in the xylem (Lang and Barrs, 1965) and, as a result, the water tension measured in the leaf can be assumed to be equal to the water tension in the branch at the base of the petiole (Richter, 1973).

### Instrument configuration

#### Cherry sapling

**Fig. 4.**
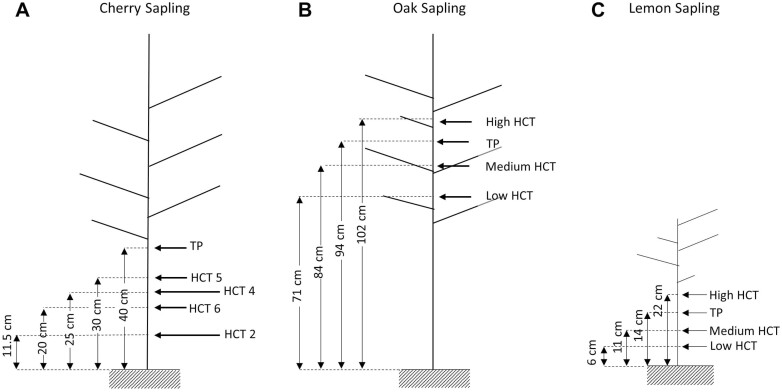
Position of instruments on the (A) cherry sapling, (B) oak sapling, and (C) lemon sapling.

The position of the instruments is shown in Fig. 4A. The HCTs and the TP on each stem were spaced by ~10 cm. The installation sites were selected to have a stem diameter wide enough to allow the installation of the instruments. There were no junctions of secondary branches between the instruments. The tensiometers HCT5 and HCT6 and the TP were installed at the beginning of the experiment. When the HCTs cavitated, they were replaced with the tensiometers HCT2 and HCT4, installed at slightly different heights.

The sapling was kept well irrigated before the experiment. During the experiment, the sapling was kept in the laboratory at constant temperature and relative humidity, close to a growth lamp to mimic solar radiation (the lamp was switched on from 06.00 h to 20.00 h and switched off from 20.00 h to 06.00 h). The sapling was allowed to enter a condition of drought over the first 18 d by stopping any irrigation. Water was then added on day 18 and on day 27. These different conditions were imposed to explore different ranges of xylem water potential. A few pressure chamber measurements were taken throughout the experiment as a reference.

#### Oak sapling

The instruments were installed with a spacing of ~10 cm (Fig. 4B), with the TP between the two HCTs. At the beginning of the experiment, two HCTs were installed at 84 cm (medium HCT) and 102 cm (high HCT) from the level of the soil. On day 13, a new HCT was installed at 71 cm (low HCT).

There were no junctions of secondary branches between the TP and the medium HCT. There was a junction of a secondary branch between these two instruments and the low and high HCTs, respectively (Fig. 4B). However, the experimental data presented in the Results showed that the low and high HCTs (positioned below and above junctions, respectively) and the medium HCT (positioned between two consecutive junctions) exhibited average differences comparable with the differences observed on the cherry and lemon saplings, where no junctions were present, as shown in Supplementary Table S3. It was therefore concluded that the presence of the junction did not affect the measurements.

The tree was not irrigated for 19 d and this generated low xylem water potential, making the HCTs more prone to cavitation. The third HCT was then added to increase the probability of having at least two active HCTs on the stem at the same time. When an HCT cavitated, it was removed for resaturation and substituted with a freshly saturated HCT. The new HCT was placed at the same installation site after removing a further layer of xylem to expose fresh xylem. Pressure chamber readings were taken approximately every 3 d during the first 14 d and daily afterwards (twice daily when the water potential was at its minimum).

Before the experiment, the oak sapling was kept in the laboratory and irrigated regularly. During the experiment, it was kept in the laboratory at constant temperature and relative humidity, close to a growth lamp to mimic solar radiation (the lamp was switched on from 06.00 h to 20.00 h and switched off from 20.00 to 06.00 h). Irrigation was stopped during the first part of the experiment to achieve drought conditions. On day 19, the soil was submerged with water and kept fully saturated until day 25. Afterwards, the water was allowed to drain freely from the bottom of the pot.

#### Lemon sapling

The instruments were installed with spacing between 3 cm and 8 cm (Fig. 4C). At the beginning of the experiment, three HCTs were installed, at 6 cm (low HCT), 11 cm (medium HCT), and 22 cm (high HCT) from the level of the soil. The TP, was installed between the medium and high HCTs at 14 cm from the soil surface.

When an HCT cavitated, it was removed, resaturated for at least 24 h, and reinstalled at the same installation site after removing a further layer of xylem to expose fresh xylem. The medium and high HCTs were removed and reinstalled on day 22 without resaturating them and only exposing fresh xylem.

The sapling was kept well irrigated before the experiment. During the experiment, the sapling was kept in the laboratory at constant temperature and relative humidity, close to a growth lamp to mimic solar radiation (the lamp was switched on from 06.00 h to 20.00 h and switched off from 20.00 h to 06.00 h). The sapling was allowed to enter a condition of drought over the first 8 d by stopping irrigation. Water was then supplied on day 8, 16, 28, 29, 32, and 35 in different amounts to explore the response of the instruments to different increments in water potential. A few pressure chamber measurements were taken throughout the experiment as a reference.

## Results

### Cherry sapling

**Fig. 5.**
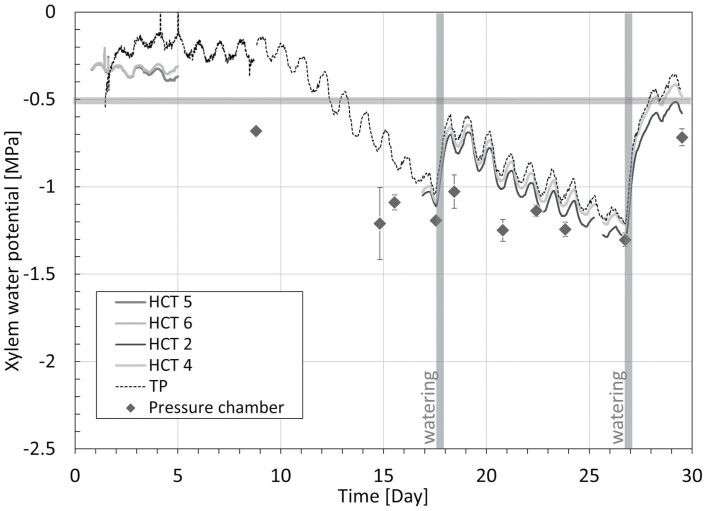
Measurement of xylem water pressure by the HCT and TP, and by the pressure chamber on non-transpiring leaves, on the cherry sapling. The measurements made with the different HCTs and the TP are indicated by the different curves. For the pressure chamber measurements, grey diamonds represent the average values and the error bars represent the SD. The vertical grey bands indicate watering times. The horizontal grey line marks the value of –0.5 MPa xylem water potential.

The measurements of xylem water potential made on the oak sapling by the HCTs and the TP are compared in Fig. 5. The measurements obtained with the pressure chamber are also shown in the same figure. Daily cycles were clearly visible in the HCT and TP continuous measurements, consistent with the cycles of light and dark imposed by the growth lamp. The xylem water potential reached its minimum around 15.00 h, when the lamp was on, and reached its maximum at around 06.00 h, when the lamp was off. The daily fluctuations were quite limited in the first 10 days of the experiment (~0.08 MPa) and larger afterwards (~0.15 MPa).

The TP measured markedly higher values of xylem water potential than the HCTs in the first 5 d (when xylem water potential was relatively high). From day 5 to day 16, only the TP measurement was available. The measurements made by the two HCTs were discarded as their readings diverged by several hundreds of kPa. This could have been due to either ongoing cavitation in one of the HCTs or a genuine difference in xylem water pressure at the two measuring sites. As it was not possible to identify the faulty measurement, both HCT measurements were discarded. TP measurement was markedly higher than that of the pressure chamber, although the difference tended to reduce when the TP readings started to decrease due to prolonged drought.

From day 16 onwards, the HCT and TP measurements were very consistent in terms of both the values measured and the response time (measurement differential was 0.065±0.021 MPa; Supplementary Table S3). The HCTs and the TP also responded promptly to watering on day 17 and day 26. HCT and TP measurements were higher than those obtained with the pressure chamber, although the difference again tended to reduce at lower xylem water potentials (from ~0.3 MPa on day 18 to <0.1 MPa on day 27).

### Oak sapling

**Fig. 6.**
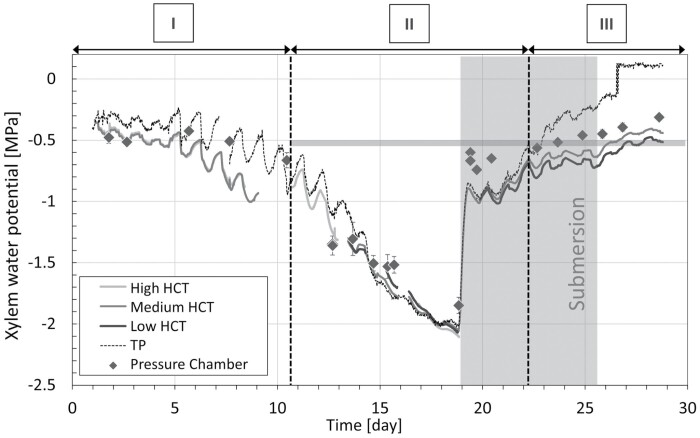
Measurement of xylem water potential by the HCT and TP, and by the pressure chamber on non-transpiring leaves, on the oak sapling. The measurements made with the different HCTs and the TP are indicated by the different curves. For the pressure chamber measurements, grey diamonds represent the average values and the error bars represent the SD of the pressure chamber measurements. The grey area indicates the period of submersion of the soil. The horizontal grey line marks the value of –0.5 MPa water potential. The vertical dotted lines separate the intervals of xylem water pressure measurement by TP above (I, III) or below (II) –0.5 MPa.

The measurements of xylem water potential made on the oak sapling via the HCTs and the TP are compared in Fig. 6. The measurements obtained with the pressure chamber are also shown in the same figure. The soil was initially well watered and was then allowed to enter drought conditions by not watering at all for the first 18 d of the experiment. The minimum xylem water potential was reached at day 18.

The soil was then submerged on day 18 and kept submerged until day 25 to release the water tension in the xylem (represented by the grey-shaded area in Fig. 6). The instruments responded promptly to the submersion on day 18, showing a sudden increase in water potential in the first 10 h following submersion. From day 19 to the end of the experiment, the xylem water potential continued to increase at a slower rate.

Daily cycles were clearly visible in both HCT and TP measurements. These fluctuations appeared to be relatively small over the first 5 d of the experiment under well-watered conditions, and between day 15 and 18 under drought conditions when the xylem water potential dropped below –1.5 MPa. The daily cycles were consistent with the cycles of light and dark imposed by the growth lamp, with maximum values of xylem water pressure recorded at around 06.00 h.

When comparing the measurements made by the HCTs and the TP, three intervals can be clearly identified in Fig. 6. During interval I, the measurement from the TP showed higher water potential values than those from the HCTs (measurement differential was 0.176±0.102 MPa; Supplementary Table S3). Measurements made by the pressure chamber were not always consistent with those from the HCTs and the TP; the pressure chamber measurements matched the HCT measurements on days 2 and 3 and the TP measurements on days 5.5. and 7.5.

During interval II, the HCTs and the TP were very consistent in terms of both values measured and response time (measurement differential was 0.028±0.045 MPa; Supplementary Table S3). In this interval, the pressure chamber measurements matched those of both the HCTs and the TP before water submersion, whereas after water submersion the pressure chamber returned water potential values higher than both the HCTs and the TP. During interval II, the water potential measured by the TP remained lower than –0.5 MPa. It should also be noted that after submersion (days 18–22), the HCTs and the TP reported very consistent values, suggesting that the measurement of xylem water potential by both instruments is reliable. Nonetheless, the water potential measured by the pressure chamber appeared to be higher than the values obtained with both the HCT and the TP.

During interval III, the water potential recorded by the TP and the HCTs deviated markedly (measurement differential 0.382±0.151 MPa; Supplementary Table S3). The TP values for water potential kept increasing until the instrument returned ‘out-of-range’ positive values. The pressure chamber measurements obtained both during and after submersion were again higher than those obtained with the HCTs. The trend of the HCT and pressure chamber measurements appeared to be very similar, as if they were both driven by the same ‘boundary condition’. The measurement by the TP followed an entirely different trend compared with the measurements made by the pressure chamber and the HCT.

### Lemon sapling

**Fig. 7.**
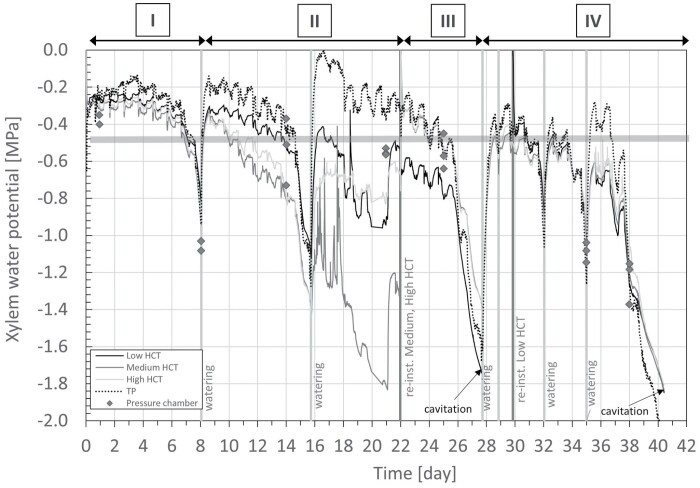
Measurement of xylem water pressure by the HCT and TP, and by the pressure chamber on non-transpiring leaves, on the lemon sapling. The measurements made with the different HCTs and the TP are indicated by the different curves. For the pressure chamber measurements, grey diamonds represent the average values and the error bars represent the SD of the pressure chamber measurements. The vertical light grey bands indicate watering; the vertical dark grey bands represent the times at which HCTs were re-installed. The thin black arrows indicate instances of cavitation occurring in the HCTs.

The measurements of xylem water potential made on the lemon sapling via the HCTs and the TP are compared in Fig. 7. The measurements obtained with the pressure chamber are also shown in the same figure. The soil was initially well watered and was then allowed to enter drought conditions by not watering at all for the first 8 d of the experiment. In total, six watering events took place during the 42 days of this experiment, on days 8, 16, 28, 29, 32, and 35. All sensors (HCTs and TC) responded immediately to the watering events.

Daily cycles were clearly visible both the HCT and the TP continuous measurements, which were consistent with the cycles of light and dark imposed by the growth lamp. The xylem water potential reached its minimum around 15.00 h, when the lamp was on, and reached its maximum at around 06.00 h, when the lamp was off.

Before the first watering on day 8 (interval I in Fig. 7), the HCT and TP measurements were consistent in terms of both values measured and response time, with the measurements from the TP being slightly higher than those from the HCTs (measurement differential was 0.081±0.030 MPa; Supplementary Table S3). These measurements were also similar to the pressure chamber measurements made on day 1 and day 8 (before watering).

After the first watering on day 8 following the drop of water potential to ~–1 MPa and the second watering on day 16 following the drop of water potential to ~–1.3 MPa (interval II), water potential measured by the TP increased much more than the water potential measured by the HCTs. This discrepancy remained over the whole of interval II with the exception of the period from day 15 to day 16, when the water potential measured by the TP dropped to values lower than –0.5 MPa (the differential in this time interval was 0.521±0.315 MPa; Supplementary Table S3). The pressure chamber measurements in interval II were lower than those obtained with the TP (consistent with transpiration-induced xylem water flow) and higher than those from the HCTs.

On day 22 (interval III), the medium and high HCTs were removed and reinstalled immediately at the same installation sites after removing a further layer of xylem to expose fresh xylem. A fresh paste of kaolin was used to establish a hydraulic connection between each HCT and the xylem water. The two reinstalled HCTs immediately recorded measurements aligned with those of the TP. The low HCT (which was not removed from the xylem and then reinstalled) measured water potential values markedly lower than the TP and the other two HCTs until their measurements dropped below –0.5 MPa on day 25. Again, as the value measured by the TP reduced below –0.5 MPa, the discrepancies between the measurements of the TP and the low HCT disappeared. The differential between the measurements made by the TP and the average measurements of the medium and high HCTs was 0.050±0.100 MPa in this time interval (Supplementary Table S3).

After watering on day 28 following the drop of water potential to ~–1.7 MPa (interval IV), the same behaviour was again observed. As the value measured by the TP reduced below –0.5 MPa, discrepancies between the measurements of the TP and HCTs essentially vanished (differential for TP <–0.5 MPa was 0.026±0.143 MPa; Supplementary Table S3). After watering on days 28, 32, and 35, with the water potential measured by the TP rising above –0.5 MPa, the discrepancies reappeared (differential for TP ≥–0.5 MPa was 0.143±0.087 MPa; Supplementary Table S3).

## Discussion

### Response time of TP and HCT

The response times of the HCT and the TP are controlled by very different mechanisms. Equilibration time is controlled by the flow of liquid water to and from the kaolin paste in the case of the HCT, whereas it is controlled by the transfer of water vapour from and to the air gap adjacent to the xylem for the TP. Since the HCT and the TP were found to respond remarkably similar to changes in boundary conditions—in particular, they responded very promptly to watering—it can be concluded that the response time of both instruments is adequate to capture hourly variations of xylem water potential. This is a major outcome of this study achieved thanks to the real-time comparison of the two instruments.

### Measurement precision of TP and HCT at low water potential values

The precision of the HCT and TP measurements needs to be discussed separately depending on whether the water potential measured by the TP is lower or higher than ~–0.5 MPa. For the case where the water potential is lower than ~–0.5 MPa, the two instruments return very similar measurements, as shown in Figs 5–7. This is a second major outcome of this study. Until now, the TP could be compared only with the pressure chamber to validate its measurements. However, the comparison between the water potential measurements made at two different sites along the transpiration-induced flow path (as is the case when comparing the TP installed on the stem and the pressure chamber with excised leaves) is not straightforward. Because water flow requires water potential gradients, a water potential differential will be established between the stem and the junction between the branch and the leaf petiole. This water potential differential is not always negligible, as shown experimentally by Dainese and Tarantino (2020) and, as a result, the pressure chamber does not represent in principle a valid measurement to benchmark the measurement of the TP. This study allowed, for the first time, assessment of the precision of the TP by benchmarking its measurements against independent measurements made at the same site in the transpiration-induced water flow path. Reciprocally, the TP allowed validation of the HCT measurements, at least in the range of water potential lower than –0.5 MPa.

### Measurement precision of TP and HCT at high water potential values

Notable discrepancies between the TP and HCT measurements generally appeared in the range where the TP measured water potentials greater than ~-0.5 MPa (see interval III in Fig. 6, and intervals I, II, and IV in Fig. 7); the question is whether the ‘faulty’ measurements are attributable to the HCT or the TP. Inspection of the measurements on the four saplings revealed that there were two exceptions, the measurement on the pear sapling (Supplementary Appendix S2) and measurement of the lemon sapling in Interval III (Fig. 7). These two sets of measurements have in common the use of kaolin paste that had never been exposed to water potential lower than the current measured value (the water potential of the paste is zero at installation). Fig. 7 also shows clearly that the difference between the HCT and the TP measurements depends on the paste, and not the HCT itself. The high and medium HCTs used on the lemon sapling at the end of interval II (where notable differences appeared between the TP and HCT measurements) are the same HCTs as those used at the beginning of interval III (where the TP and HCT measurements are remarkably similar). The difference between the two intervals is the lowest water potential ever experienced by the kaolin paste. In interval II, the paste was brought to water potentials lower than –1 MPa before its water potential increased again due to watering. In interval III, where a new kaolin paste was applied, the paste had never experienced a water potential lower than the value currently being measured. This suggests that the hydraulic history of the kaolin paste plays a role in the accuracy of measurements made by the HCTs. The water retention behaviour of kaolin initially prepared from a slurry state was investigated by Tarantino (2009) and is shown in Supplementary Fig. S3. When drying the paste from its slurry state, the paste remains fully saturated until a water potential of ~–1 MPa (air-entry value) is reached. In this range, the paste is efficient in transmitting water potential and this explains the good match of the HCT and TP measurements on the pear sapling (Supplementary Appendix S2) and the measurements on the lemon sapling in Interval III (Fig. 7). If the paste is dried out, that is, it experiences water potentials lower than the air-entry value (–1 MPa), the paste desaturates. This does not prevent the transmission of water potential, as shown in interval III of Fig. 7 where the TP and HCT measurements match fairly well. Upon rewetting, the kaolin never recovers full saturation due to the air cavities remaining occluded in the pore space. Remarkably, the air-occlusion value of –0.5 MPa matches the xylem water potential at which discrepancies were observed between the TP and HCT measurements. It can therefore be concluded that if the paste first experiences a water potential lower than its air-entry value (i.e. the paste desaturates) and the water potential then increases again to values higher than –0.5 MPa, air cavities remain occluded in the paste and this hampers the proper transmission of the water potential.

The measurements made by the HCT in the range from 0 to –0.5 MPa are therefore probably not reliable. At the same time, concerns also arise about the measurements made by the TP in the range from 0 to –0.5 MPa. The measurements made by the HCT at the beginning of the experiments on the cherry and oak saplings (Figs 5, 6) should not be affected by the fresh paste applied to the HCT, and the discrepancies in the measurements recorded may be due to the TP rather than the HCT. Furthermore, the positive values returned by the TP at the end of the experiment on the oak sapling (Fig. 6) also seem to suggest that the TP might be not very accurate in this range. However, no clear conclusions can be drawn, and further investigation is required to address this issue.

### Comparison of the TP and HCT against the pressure chamber

**Fig. 8.**
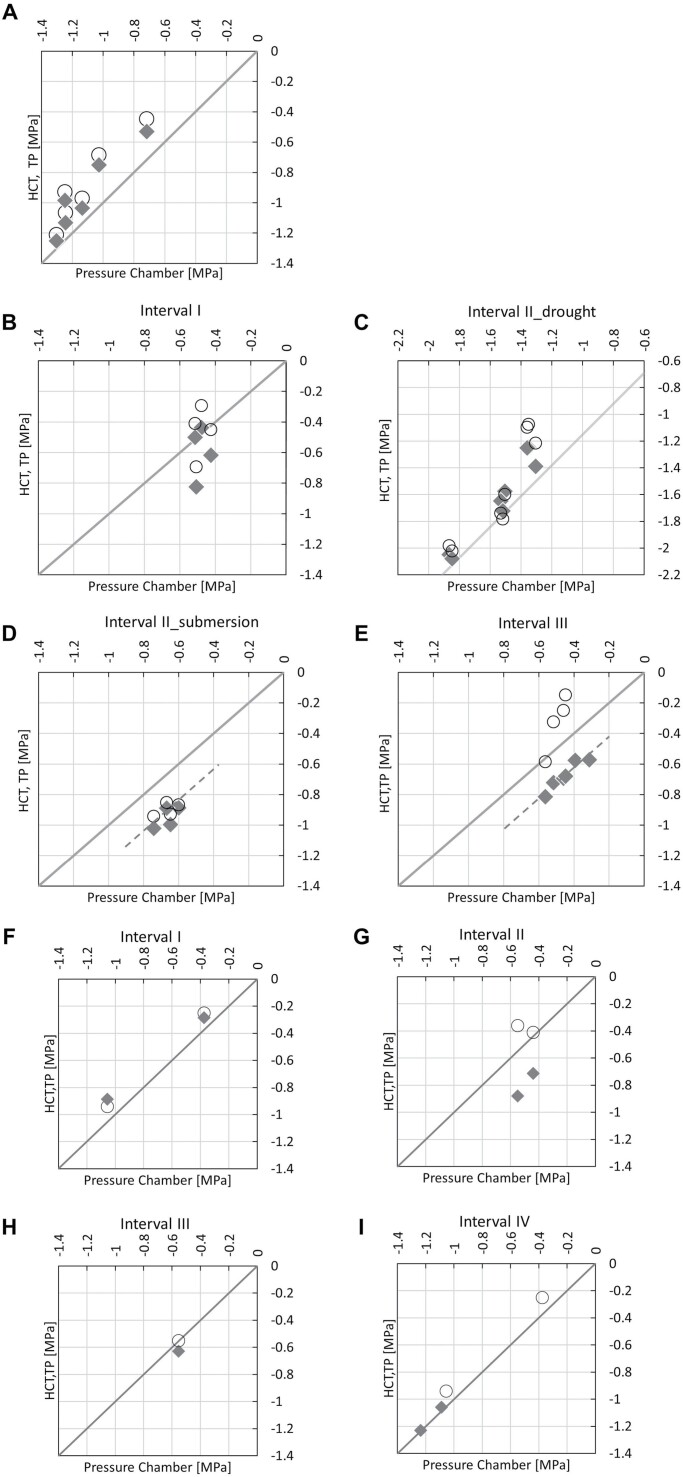
Comparison of xylem water potential measured by the pressure chamber (horizontal axis) versus the TP (open circles) and the HCT (solid diamonds) measured on the (A) cherry sapling; (B) oak sapling during interval I; (C) oak sapling during interval II drought; (D) oak sapling during interval II submersion; (E) oak sapling during interval III; (F) lemon sapling during interval I; (G) lemon sapling during interval II; (H) lemon sapling during Interval III; and (I) lemon sapling during interval IV.

The TP and HCT can be further investigated by benchmarking their measurements against the measurements made by the pressure chamber. The good agreement between the HCT and TP at water potentials (as recorded by the TP) lower than ~–0.5 MPa is also evident in Fig. 8A, C, D, F, and I. The HCT and TP generally return values of water potential higher than those measured by the pressure chamber, which is consistent with the direction of transpiration-induced sap flow. The discrepancy tends to reduce at lower values of water potential, which is also intuitive. The transpiration is likely to enter a water-limited condition in this range, that is, stomata partially close to reduce transpiration, and this generates a smaller water potential differential between the leaf and the stem. Overall, these figures show that the values measured by the pressure chamber on excised leaves can be markedly lower than the water potential measurements made at the stem (by the TP and HCT), and the pressure chamber should therefore be used with care to validate TP or HCT measurements. It should also be noted that the water potential measured by the pressure chamber was higher than both HCT and TC measurements after submersion of the soil in the oak sapling experiment during interval II (Fig. 8D). This might be associated with the leaves entering a state of anaerobiosis, but a discussion of the processes leading to this reversed water potential differential between the leaves and stem is outside the scope of this paper. However, Fig. 8D again confirms that the pressure chamber measurement may not always be considered as a reference to validate either TP or HCT measurements.

In the low water potential range (Fig. 8B, E, G), the pressure chamber does not seem to support either the TP or HTC measurements. Again, further studies should be carried out to investigate the precision of the measurements made by the HCT and TP at high water potentials.

### Conclusions

This work has cross-validated two different instruments for the continuous non-destructive measurement of xylem water potential, the HCT and the TP. The HCT and the TP were found to respond remarkably in phase to changes in boundary conditions, in particular to watering, despite very different working principles. It was concluded that the response time of both instruments is adequate to capture hourly variations of xylem water potential; this is a major outcome of this study achieved thanks to the real-time comparison of these two instruments.

The HCT and the TP returned very similar xylem water potential measurements for water potential values <~–0.5 MPa (differences were typically lower than 0.10–0.15 MPa). Again, as the working principles of the two instruments are very different, these measurements made it possible to demonstrate that the HCT and the TP show satisfactory accuracy in this range of xylem water potentials. Previously, the TP could be compared only with the pressure chamber to validate its measurements. However, the water potential at the leaf (specifically, the junction between the leaf petiole and the branch) can be markedly different from the water potential at the stem, as demonstrated in this study and as expected theoretically (as transpiration-induced water flow requires a non-zero water potential differential between the stem and leaves). As a result, this study has provided, for the first time, a robust assessment of the TP by benchmarking its measurements against an independent measurement made at the same site in the transpiration-induced water flow path.

At water potential values higher than ~–0.5 MPa, the measurements made by the HCT may be affected by the kaolin paste used to make contact between the porous ceramic filter of the HCT and the xylem. If the kaolin paste is subjected to water potentials lower than its air-entry values (~–1 MPa), it undergoes desaturation. If rewetting is associated with water potentials higher than its air-occlusion value (~–0.5 MPa), air cavities may remain trapped in the paste, hampering the transmission of water potential from the xylem to the HCT. Entrapped air cavities play a role only if the paste is subjected first to drying and then to rewetting (due to hydraulic hysteresis). This problem did not appear if the kaolin paste was subjected to a current water potential that was the lowest it had ever experienced (monotonic drying path). This is a current limitation of the HCT that could be overcome by selecting a clay paste with an enhanced air-entry value. For example, the London clay reconstituted from slurry tested by Marinho (1994) showed an air-entry value of the order of 10 MPa, and this would have remained saturated under the water potentials investigated in this work. At water potentials higher than ~–0.5 MPa, the measurements made by the TP also presented some inconsistencies, which would require further studies to investigate.

Finally, this cross-validation was been carried out in the laboratory at 20 °C (the HCT and TP were also calibrated at the same temperature). In the field, temperature can vary markedly and the performance of these two instruments can also vary markedly. To investigate the effects of temperature, the TP and the HCT were calibrated in the laboratory at different temperatures. It was shown that the effect of temperature on the measurements made by the HCT is negligible (error <0.03 MPa), whereas it becomes important for the TP, which showed errors of up to 0.66 MPa when the temperature varied from 20 °C to 40 °C.

## Supplementary data

The following supplementary data are available at *JXB* online.

Table S1. Molality, relative humidity, and water potential at 20 °C according to Lang (1967) and Romero (1999).

Table S2. Characteristics of the saplings selected for the test.

Table S3. Measurement errors.

Fig. S1. Instruments installed on the saplings.

Fig. S2. HCT installation on the stem.

Fig. S3. Main drying and main wetting water retention curve of the kaolin used to make the contact paste for the HCT.

Appendix S1. Comparison between measured and theoretical water potential.

Appendix S2. Supplementary test on pear sapling.

erab412_suppl_Supplementary_Tables_S1-S3_Figures_S1-S3_Appendix_S1-S2

## Data Availability

The data supporting the findings of this study are available from the corresponding author, Alessandro Tarantino, upon request.

## References

[CIT0001] Balling A, Zimmermann U. 1990. Comparative measurements of the xylem pressure of Nicotiana plants by means of the pressure bomb and pressure probe. Planta 182, 325–338. 24197182 10.1007/BF02411382

[CIT0002] Boyer JS . 1995. Measuring the water status of plants and soils. London: Academic Press

[CIT0003] Bulut R, Leong E. 2008. Indirect measurement of suction. Geotechnical and Geological Engineering 26, 21–32.

[CIT0004] Campbell G, Gardner W. 1971. Psychrometric measurement of soil water potential:temperature and bulk density effect. Soil Science Society of America Journal 35, 8–12.

[CIT0005] Charrier G, Burlett R, Gambetta G, Delzon S, Domec J-C, Beaujard F. 2017. Monitoring xylem hydraulic pressure in woody plants. Bio-Protocol 7, e2580. 34595262 10.21769/BioProtoc.2580 PMC8438495

[CIT0006] Dainese R, Tarantino A. 2020. Measurement of plant xylem water pressure using the high-capacity tensiometer and implications on the modelling of soil-atmosphere interaction. Géotechnique 71, 441–454.

[CIT0007] De Benedetti P . 1996. Metastable liquids. Princeton: Princeton University Press.

[CIT0008] Dixon MA, Tyree MT. 1984. A new stem hygrometer, corrected for temperature-gradients and calibrated against the pressure bomb. Plant, Cell and Environment 7, 693–697.

[CIT0009] Goode J, Higgs K. 1973. Water, osmotic and pressure potential relationships in apple leaves. Journal of Horticultural Science 48, 203–215.

[CIT0010] ICT International . 2017. Calibration. http://ictinternational.com/content/uploads/2014/03/PSY-Calibration.pdf. Accessed May 2021.

[CIT0011] ICT International . 2021. Psychrometer PSY1 Manual. http://ictinternational.com/products/psy1/psy1-stem-psychrometer. Accessed May 2021.

[CIT0012] Jones HG . 2006. Monitoring plant and soil water status: established and novel methods revisited and their relevance to studies of drought tolerance. Journal of Experimental Botany 58, 119–130. 16980592 10.1093/jxb/erl118

[CIT0013] Lang A, Barrs H. 1965. An apparatus for measuring water potential in the xylem of intact plants. Australian Journal of Biological Sciences 18, 487–497.

[CIT0014] Lang ARG . 1967. Osmotic coefficients and water potentials of sodium chloride solutions from 0 to 40 C. Australian Journal of Chemistry 20, 2017 – 2023.

[CIT0015] Marinho FAM . 1994. Shrinkage behaviour of some plastic soils. PhD thesis, Imperial College London.

[CIT0016] Marinho FAM, Take WA, Tarantino A. 2008. Measurement of matric suction using tensiometric and axis translation techniques. Geotechnical and Geological Engineering 26, 615–631.

[CIT0017] Martinez E, Cancela J, Cuesta T, Neira X. 2011. Review. Use of psychrometers in field measurements of plant material: accuracy and handling difficulties. Spanish Journal of Agricultural Research 9, 313–328.

[CIT0018] Mendes J, Gallipoli D, Boeck F, Tarantino A. 2020. A comparative study of high capacity tensiometer designs. Physics and Chemistry of the Earth, Parts A/B/C. 120, 102901.

[CIT0019] Patankar R, Quinton WL, Baltzer JL. 2013. Permafrost-driven differences in habitat quality determine plant response to gall-inducing mite herbivory. Journal of Ecology 101, 1042–1052.

[CIT0020] Richter H . 1973. Frictional potential losses and total water potential in plants: a re-evaluation. Journal in Experimental Botany 24, 983–994.

[CIT0021] Ridley A, Burland JB. 1993. A new instrument for the measurement of soil moisture suction. Géotechnique 43, 321–324.

[CIT0022] Romero E . 1999. Thermo-hydro-mechanical behaviour of unsaturated Boom clay: an experimental study. PhD Thesis, Universidad Politècnica de Catalunya.

[CIT0023] Tarantino A . 2004. Panel lecture: direct measurement of soil water tension. In: Juca JFT, de Campos TMP, Marinho FAM, eds. Third International Conference on Unsaturated Soils. Recife, Brazil 2002 Proceedings. Rotterdam: Balkema, 1005–1017.

[CIT0024] Tarantino A . 2009. A water retention model for deformable soils. Géotechnique 59, 751–762.

[CIT0025] Tarantino A, Mongioví L. 2001. Experimental procedures and cavitation mechanisms in tensiometer measurements. Geotechnical and Geological Engineering 19, 189–210.

[CIT0026] Tarantino A, Mongioví L. 2002. Design and construction of a tensiometer for direct measurement of matric suction. In: Juca JFT, de Campos TMP, Marinho FAM, eds. Third International Conference on Unsaturated Soils. Recife, Brazil 2002 Proceedings. Rotterdam: Balkema, 319–324.

[CIT0027] Tarantino A, Mongioví L. 2003. Calibration of tensiometer for direct measurement of matric suction. Géotechnique 53, 137–141.

[CIT0028] Wang H, Guan H, Deng Z, Simmons C. 2014. Optimization of canopy conductance models from concurrent measurements of sap flow and stem water potential on Drooping Sheoak in South Australia. Water Resources Research 50, 6154–6167.

[CIT0029] Wei C, Steudle E, Tyree M, Lintilhac P. 2001. The essentials of direct xylem pressure measurement. Plant, Cell and Environment 24, 549–555.

[CIT0030] Yang YT, Guan HD, Hutson JL, Wang HL, Ewenz C, Shang SH, Simmons CT. 2013. Examination and parameterization of the root water uptake model from stem water potential and sap flow measurements. Hydrological Processes 27, 2857–2863.

