# Supplementary material for: Cross-validation of the high-capacity tensiometer and thermocouple psychrometer for continuous monitoring of xylem water potential in saplings
Source: J Exp Bot. 2021 Sep 10;73(1):400–12. doi: 10.1093/jxb/erab412 (PMC8730697; doi:10.1093/jxb/erab412)
Supplement: erab412_suppl_Supplementary_Tables_S1-S3_Figures_S1-S3_Appendix_S1-S2 [file erab412_suppl_supplementary_tables_s1-s3_figures_s1-s3_appendix_s1-s2.pdf]

Cross-validation on saplings of High-Capacity Tensiometer and Thermocouple Psychrometer for continuous monitoring of xylem water potential

Supplementary data

Supplementary Tables

Table S1. Molality, relative humidity (RH), and water potential at 20℃ according to Lang (1967) and Romero (1999) (see Appendix S1) of the six solutions of NaCl used for the calibration of the thermocouple psychrometer

	Lang (1967)		Romero (1999), see Appendix	
Molality, m	RH	Water potential [MPa]	RH	Water potential [MPa]
0.1	0.996	-0.454	0.997	-0.429
0.2	0.993	-0.900	0.994	-0.862
0.3	0.990	-1.344	0.990	-1.300
0.4	0.987	-1.791	0.987	-1.742
0.5	0.983	-2.241	0.984	-2.189
1	0.967	-4.550	0.967	-4.494

Table S2. Characteristics of the saplings selected for the test.

	Cherry tree	Oak tree	Pear tree	Lemon tree
Total height [m]	1.90	2.30	1.50	0.55
Diameter at 100 mm from the soil [mm]	24	21	25	18

Table S3. Measurement errors

Cherry sapling (Figure 5)	Days 1-5		HCT6 minus HCT5 [MPa]	TP minus $HCT_{average}$ [MPa]
		Average	0.018	0.139
		Stan. Dev	0.017	0.049
	Days 17-28		HCT2 minus HCT4 [MPa]	TP minus $HCT_{average}$ [MPa]
		Average	0.058	0.065
		Stan. Dev	0.017	0.021
Oak sapling (Figure 6)	Days 1-8 (Interval I)		HCT_{Medium} minus HCT_{High} [MPa]	TP minus $HCT_{average}$ [MPa]
		Average	0.005	0.176
		Stan. Dev	0.014	0.102
	Days 17-19 / 20-22 (Interval II)		HCT_{Medium} minus HCT_{Low} [MPa]	TP minus $HCT_{average}$ [MPa]
		Average	0.013	0.028
		Stan. Dev	0.032	0.045
	Days 22-28 (Interval III)		HCT_{Medium} minus HCT_{Low} [MPa]	TP minus $HCT_{average}$ [MPa]
		Average	0.073	0.382
		Stan. Dev	0.015	0.151
Pear sapling (Figure S4)	Days 2-7		HCT3 minus HCT4 [MPa]	TP minus $HCT_{average}$ [MPa]
		Average	0.032	0.012
		Stan. Dev	0.013	0.036
Lemon sapling (Figure 7)	Days 1-8 (Interval I)		HCT_{Medium} minus HCT_{High} [MPa]	TP minus $HCT_{average}$ [MPa]
		Average	0.024	0.081
		Stan. Dev	0.011	0.030
	Days 8-22 (Interval II)		HCT_{Medium} minus HCT_{High} [MPa]	TP minus $HCT_{average}$ [MPa]
		Average	0.312	0.521
		Stan. Dev	0.351	0.315
	Days 22-28 (Interval III)		HCT_{Medium} minus HCT_{High} [MPa]	TP minus $HCT_{average}$ [MPa]
		Average	0.000	0.050
		Stan. Dev	0.009	0.100
	Days 28-40 (Interval IV, TP<-0.5 MPa)		HCT_{Medium} minus HCT_{High} [MPa]	TP minus $HCT_{average}$ [MPa]
		Average	0.026	0.026
		Stan. Dev	0.040	0.143
	Days 28-40 (Interval IV, TP≥-0.5 MPa)		HCT_{Medium} minus HCT_{High} [MPa]	TP minus $HCT_{average}$ [MPa]
		Average	0.017	0.143
		Stan. Dev	0.023	0.087

Supplementary Figures

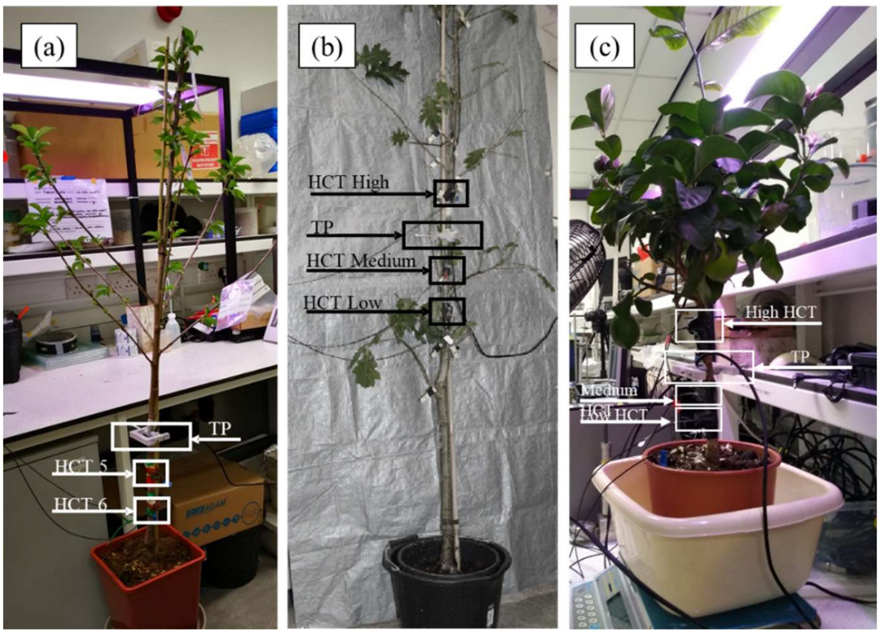

Fig. S1. Instruments installed on the (a) cherry sapling, (b) oak sapling, and (c) lemon sapling

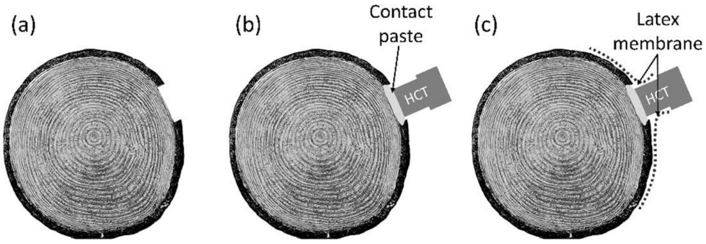

Fig. S2. HCT installation on stem. (a) exposure of xylem tissues. (b) HCT application. (c) sealing with latex membrane

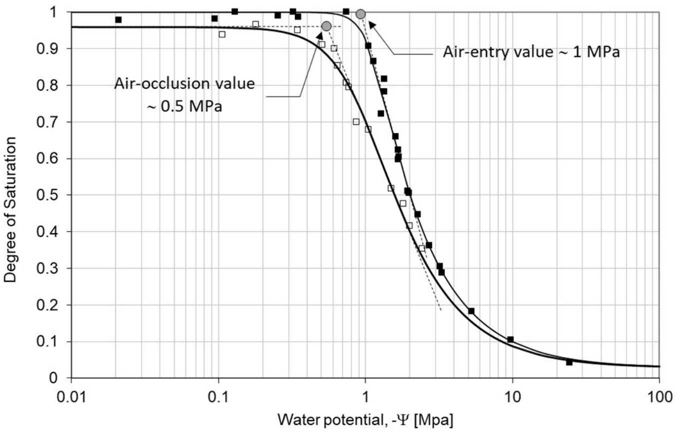

Fig. S3. Main drying and main wetting water retention curve of the kaolin used to make the contact paste for the HCT (after Tarantino, 2009)

Appendix S1: Comparison between measured and theoretical water potential

Water potential imposed by NaCl aqueous solution at given temperature and molality was derived via the psychometric law:

$$\Psi = -\frac{RT}{\frac{M_w}{\rho_w}}\ln(RH) \qquad [1]$$

where RH is the relative humidity, R is the universal gas constant (R=8.314 J·K^{-1}·mol^{-1}), T the absolute temperature, M_w is the molecular mass of pure water (M_w=18.015 kg/kmol), and ρ_w is the density of pure water.

In turn, the relative humidity was assumed to be dependent on the molality m of the NaCl aqueous solution according to Romero (1999):

$$\begin{aligned} &\mathrm{RH} = 1 - 0.035\,m - 1.1421\,\cdot 10^{-3}\,m(m-3) && m < 3\ \mathrm{mol/kg} \\ &\mathrm{RH} = 1 - 0.035\,m - m(m-3)(1.9772\cdot 10^{-3} - 1.193\cdot 10^{-5}T) && m \geq 3\ \mathrm{mol/kg} \end{aligned} \qquad [2]$$

Water density ρ_w was assumed to depend on temperature T according to the following empirical equation developed by Romero (1999) by fitting experimental data by Batchelor (1983) and Perry (1992)

$$\rho_w = 1007.9\exp[-4.573\cdot 10^{-4}(T - 273.15)] \qquad [3]$$

with ρ_w in kg/m^3.

Water potential can be derived by combining Eqs. [1], [2], and [3]

Appendix S2: Supplementary Test on Pear Sapling

The position of the two HCTs and PSY1 is shown in Fig. S4. There were no junctions of secondary branches between the instruments. All instruments were installed at the beginning of the test. This pear sapling was acquired in the beginning of October. As a result of the autumn season, its leaves were already turning yellow and were falling at the touch.

The absence of transpiration and, hence, of the active control of xylem water potential was considered an interesting case study to examine. As the xylem water potential remains passively controlled by the water potential of the soil, we purposely generated evaporation from the soil to investigate whether the HCTs and the TP still respond consistently to passively induced changes in xylem water potential.

To this end, on the second day after installation of the instruments, 5 cm of topsoil was removed from the vase and a fan was placed directed to the newly exposed soil surface to accelerate the drying process. On the fourth day after installation two additional HCT were installed in the soil, one on the surface (HCT-SS) and the other one 3 cm below the soil surface (HCT-S3). No irrigation took place during the course of experiments on this sapling. It was clearly not possible to perform Pressure Chamber measurements on the leaves.

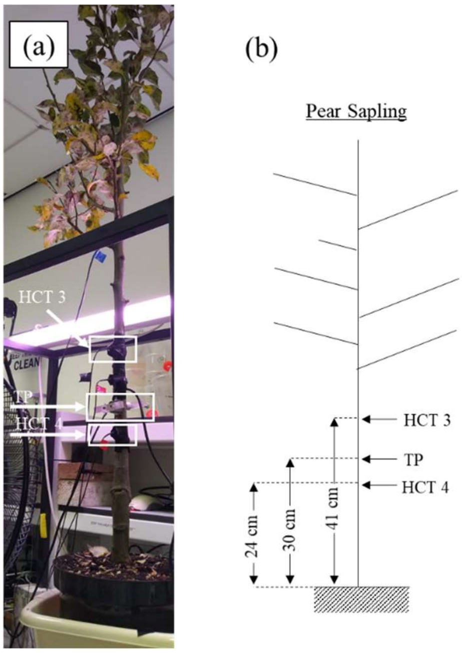

Fig. S4. Instruments (a) Installation and (b) Position on the pear sapling

The measurements of xylem water potential on the pear tree via the HCT and the TP are compared in Fig. S5. The xylem water potential measured by the HCTs reached an equilibrium a few hours after installation. It took slightly longer for the TP measurements to catch up, but it eventually did at around 1 day.

Two days after installation, a 5 cm layer of topsoil was removed from the vase and a fan was placed directed to the newly exposed soil surface to accelerate the drying process. This triggered a decay in water potential measured by the instruments that was observed from day 4 onward.

To corroborate the assumption that xylem water potential was passively driven by the decay of soil water potential, two HCT were installed four days after installation: one on the soil surface (HCT-SS) and the other one 3cm below the soil surface (HCT-S3). The water potential measured by the HCT-SS on the soil surface decreased rapidly until it cavitated around 24 h after its installation. However, the measurements of the HCT-S3 installed 3 cm below the soil surface indicated that the soil water potential decayed at a slower rate and consistent with the rate measured by the HCTs and TP. Overall, the HCTs and the TP exhibited a very consistent response despite the scattering of the TP measurements (measurement differential was 0.012±0.036 MPa, Table S3).

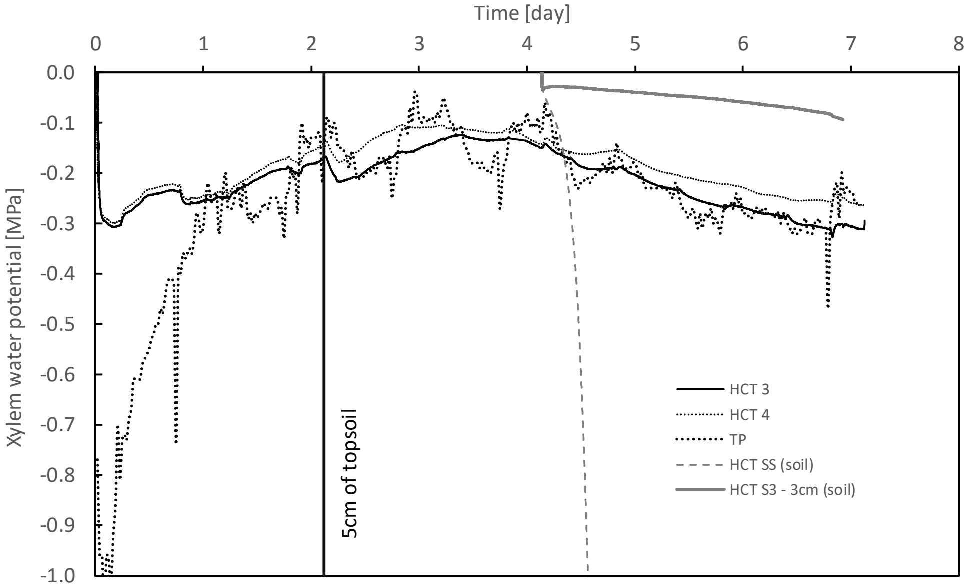

Fig. S5. Measurement of xylem water pressure via the High Capacity Tensiometer (HCT) and the Thermocouple Psychrometer (TP) on the Pear sapling. The vertical red band indicate the removal of 5 cm of topsoil.
